# Thermal and Oxygen Flight Sensitivity in Ageing *Drosophila melanogaster* Flies: Links to Rapamycin-Induced Cell Size Changes

**DOI:** 10.3390/biology10090861

**Published:** 2021-09-02

**Authors:** Ewa Szlachcic, Marcin Czarnoleski

**Affiliations:** Institute of Environmental Sciences, Faculty of Biology, Jagiellonian University, Gronostajowa 7, 30-387 Kraków, Poland; ewa.szlachcic@doctoral.uj.edu.pl

**Keywords:** body size, cell size, *Drosophila melanogaster*, flight performance, oxygen limitation, temperature, thermal limits, thermal optima, thermal sensitivity, wing load

## Abstract

**Simple Summary:**

Cold-blooded organisms can become physiologically challenged when performing highly oxygen-demanding activities (e.g., flight) across different thermal and oxygen environmental conditions. We explored whether this challenge decreases if an organism is built of smaller cells. This is because small cells create a large cell surface, which is costly, but can ease the delivery of oxygen to cells’ power plants, called mitochondria. We developed fruit flies in either standard food or food with rapamycin (a human drug altering the cell cycle and ageing), which produced flies with either large cells (no supplementation) or small cells (rapamycin supplementation). We measured the maximum speed at which flies were flapping their wings in warm and hot conditions, combined with either normal or reduced air oxygen concentrations. Flight intensity increased with temperature, and it was reduced by poor oxygen conditions, indicating limitations of flying insects by oxygen supply. Nevertheless, flies with small cells showed lower limitations, only slowing down their wing flapping in low oxygen in the hot environment. Our study suggests that small cells in a body can help cold-blooded organisms maintain demanding activities (e.g., flight), even in poor oxygen conditions, but this advantage can depend on body temperature.

**Abstract:**

Ectotherms can become physiologically challenged when performing oxygen-demanding activities (e.g., flight) across differing environmental conditions, specifically temperature and oxygen levels. Achieving a balance between oxygen supply and demand can also depend on the cellular composition of organs, which either evolves or changes plastically in nature; however, this hypothesis has rarely been examined, especially in tracheated flying insects. The relatively large cell membrane area of small cells should increase the rates of oxygen and nutrient fluxes in cells; however, it does also increase the costs of cell membrane maintenance. To address the effects of cell size on flying insects, we measured the wing-beat frequency in two cell-size phenotypes of *Drosophila melanogaster* when flies were exposed to two temperatures (warm/hot) combined with two oxygen conditions (normoxia/hypoxia). The cell-size phenotypes were induced by rearing 15 isolines on either standard food (large cells) or rapamycin-enriched food (small cells). Rapamycin supplementation (downregulation of TOR activity) produced smaller flies with smaller wing epidermal cells. Flies generally flapped their wings at a slower rate in cooler (warm treatment) and less-oxygenated (hypoxia) conditions, but the small-cell-phenotype flies were less prone to oxygen limitation than the large-cell-phenotype flies and did not respond to the different oxygen conditions under the warm treatment. We suggest that ectotherms with small-cell life strategies can maintain physiologically demanding activities (e.g., flight) when challenged by oxygen-poor conditions, but this advantage may depend on the correspondence among body temperatures, acclimation temperatures and physiological thermal limits.

## 1. Introduction

At present, the Earth’s atmosphere contains 21% O_2_ and the global average surface temperature is ca. 15 °C [1], but on a geological timescale, these parameters have been changing dramatically, driving ecological and evolutionary transitions in life. In ectotherms, global atmospheric processes are often indicated to be an important selective driver in the evolution of body size [2,3,4,5], which is the primary life history trait with strong correspondence to fitness [6,7,8]. For example, the spectacular emergence and subsequent disappearance of giant insects during the Carboniferous, Permian, and Triassic periods was linked to shifts in the amount of oxygen in the Earth’s atmosphere [2,9]. Dudley [10] suggested that the enrichment of the atmosphere with oxygen during the Phanerozoic period could have independently triggered the evolution of actively flying insects and vertebrates (Pterosaurs, birds and bats). Insects ultimately became the most numerous and diverse group of animals on Earth, which can be at least partially attributed to their efficient gas-exchange system and flight ability [3]. Extant insects show various adaptations that allow them to thrive in highly metabolically demanding environments, spanning broad gradients of thermal and oxygen conditions. For example, some insects constantly occupy hypoxic microenvironments in organic soil, burrows, grain stores or water [11], or, as seen in species of fruit flies found at 5000 m a.s.l. in the Himalayas, inhabit high elevations with oxygen-poor conditions and low temperatures [12]. We note that the high-elevation environments are characterized not only by spatial co-gradients of elevation, temperature and oxygen partial pressure, but also by dramatic temporal environmental fluctuations that can occur within hours, in addition to daily or seasonal fluctuations [12,13].

Like many other environmental conditions, ambient temperature and air oxygen levels affect many aspects of the physiology and behavior of insects in a complex way [5,14]. Such effects should not be viewed as the result of independent impacts of each factor, but rather in light of their combined effects on organismal performance and, ultimately, on Darwinian fitness. Environmental temperature and oxygen partial pressure together determine the balance between the metabolic demand for oxygen and the supply of oxygen experienced by an ectotherm. For example, the same oxygen partial pressure can provide a rich or poor oxygen supply depending on the metabolic demand dictated by body temperature and organismal activity [15]. Moreover, an increase in environmental temperatures increases the physiological rates of ectotherms, thus increasing the metabolic demand of cells for oxygen, together with the capacity to deliver oxygen to the mitochondria (ventilation, circulation and diffusion). Nevertheless, the effects of temperature on metabolic demand can exceed those on oxygen delivery [14], creating a mismatch between the demand for oxygen and the oxygen supply [16,17,18]. We can expect different ectotherm taxa to face unequal risks imposed by such mismatches depending on taxon-specific characteristics, although this topic has not been well studied. For example, terrestrial insects are often considered to be the least oxygen-limited invertebrates [12] because of their efficient tracheal gas exchange system [19,20]. Nevertheless, whether an ectotherm can meet its oxygen demand via the oxygen supply also strongly depends on its activity [15] and, thus, on the energetic requirements of biochemical pathways involved in different types of organismal activities. In a resting insect, limitation imposed by the oxygen supply is observed during prolonged exposure to oxygen air concentrations below 6% [21], while in a flying insect, an acute decrease in the air oxygen concentration to 10% is sufficient to impede flight performance [22]. A better understanding of the environmental limitations on flying insects is important, as flight performance has strong connections with Darwinian fitness. Flight confers many selective advantages by allowing insects to disperse [23,24] and, thus, find food, oviposition sites or mates or to escape from attacking predators [12,25]. On the other hand, insect flight is extremely ATP-demanding [26]. Insect flight muscles are entirely aerobic [27], and thus, their performance strongly depends on the oxygen supply. Therefore, a flying insect requires rapid ATP generation by flight muscle mitochondria and, thus, sustainable oxygen delivery to muscle cells, suggesting that flight may be more prone to oxygen limitation than other insect activities.

Like insect flight muscles, each tissue of an organism performs specific functions that require specific characteristics of cells, including organellular contents, cell shape and cell size [28]. Here, we hypothesize that the cellular architecture of tissue and organs can help ectotherms meet their metabolic demands via the oxygen supply in response to environmental conditions and organismal activity. Following Antoł et al. [29], our study uses a conceptual framework integrating earlier hypotheses about fitness costs and benefits associated with cell size differences among organisms that we refer to as the theory of optimal cell size (TOCS) ([30,31,32,33,34,35,36,37,38,39,40,41,42]; see also a recent review by Kozłowski et al. [43]). According to TOCS, the life history strategies of organisms involve different developmental cellular mechanisms that ultimately decide whether a body consists of many small cells or fewer large cells, which should have vital effects on physiology. A large-cell body confers advantages in the form of low tissue maintenance metabolic costs at the expense of a decreased capacity to supply individual cells with oxygen and nutrients, especially in the face of increased metabolic demands. All else being equal, large cells have a relatively small surface area (plasma or cell membrane) compared to small cells. In effect, large cells represent a “frugal physiology” that reduces the costliness of the molecular work aimed at maintaining ionic gradients at the cell surface and the physical state of the cell membrane [44,45,46], while handicapping the cells in terms of their transmembrane transport capacity. This type of physiology would be favored whenever the reduction of maintenance costs brings selective advantage. On the other hand, small-cell bodies represent a “wasteful physiology” that imposes high basal costs of tissue maintenance but enables rapid oxygen and resource delivery to the cell interior. This type of physiology would, for example, help ectotherms meet metabolic challenges, such as those imposed by increased temperatures coupled with a poor oxygen supply in the environment. Interestingly, the cellular composition of a body is also considered to affect the number and density of transcription sites (cell nuclei) in organs [47], molecular crowding in the cytosol [32] and the access of molecules to reaction sites inside cells [17], which would correspond to the anabolic capacity of cells [32] or the tolerance of developmental noise driven by molecular processes [17]. Ultimately, the degree to which each type of physiology, and thus the cellular composition of a body, may be favored by natural selection will depend on the balance between the supply (oxygen, nutrients) and the metabolic demand (ATP, organic compounds) of an organism in its environment [31].

To examine the predictions of TOCS regarding the physiological consequences of cell size, we studied flight performance in tethered *Drosophila melanogaster* fruit flies during exposure to an acute increase in temperature and differences in oxygen availability. The studied flies had two different cell-size phenotypes, which we initially obtained by rearing larvae on either standard food (control flies) or food supplemented with rapamycin (rapamycin flies). Rapamycin is used in human pharmacotherapy as an antibiotic and immunosuppressive drug; biochemically, it inhibits the activity of nutrient-sensing protein kinases in the TOR (target of rapamycin) signaling pathway [48,49]. The TOR pathway is evolutionarily conserved in all eukaryotes and plays a pivotal role in regulating cellular processes such as cell growth and proliferation, cytoskeleton dynamics, protein synthesis, metabolism, survival and autophagy [50,51,52,53,54]. Consequently, TOR activity is regarded as one of the major signaling pathways that controls cell size in tissues and organs at an organism-wide level [55,56], as supported by studies that have manipulated TOR activity to produce phenotypes with different cell sizes [57,58,59]. In our study, we used rapamycin supplementation to alter TOR activity in developing *D. melanogaster* larvae. After obtaining adult flies from our rearing treatments, we estimated the size of the wing epidermal cells, expecting that the control flies would exhibit phenotypes characterized by larger cells than the rapamycin flies. Then, we used our flies with two different cell-size phenotypes to measure the maximum wing-beat frequency in tethered flies originating from two age classes characterized by a 2.5-fold age difference. Following previous studies of ageing *D. melanogaster* [60], we treated our two age groups as representatives of either young or middle-aged flies, while the other group was expected to already show symptoms of age-dependent deterioration in organismal performance. Interestingly, previous studies of insects have not reported consistent age effects on flight performance [22,61,62,63,64,65,66,67]). Our flight measurements were carried out immediately after exposing flies to a sudden temperature shift either to warm (24 °C) or hot (29 °C) conditions, and these two thermal conditions were simultaneously combined with either normoxic or hypoxic conditions. Thus, the flies had to perform a highly metabolically demanding activity (flight) under different balances between oxygen demand and supply. Our study design is relevant to the ecological context, in which environmental fluctuations can challenge flies with sudden heat waves, thus changing their body temperature, either mildly within thermal optima or more severely if body temperature approaches physiological thermal limits. Generally, we expected that warmer conditions would increase the wing-beat rate, but that this flight performance measure would deteriorate under oxygen-poor conditions (hypoxia), indicative of physiological limitations caused by a poor oxygen supply to mitochondria with increased demands. Given the predictions of TOCS about cell-size effects on physiological performance, we expected a weaker limitation of flight performance by hypoxia in the small-cell vs. large-cell phenotype.

## 2. Materials and Methods

### 2.1. Flies

The studied flies originated from a wild population of *D. melanogaster* from the winery of Jagiellonian University (JU) in Krakow (south Poland). In September 2017, field-collected females were transferred to a JU laboratory to establish genetic lines (isolines) for the experiment. All laboratory flies were maintained in a thermal cabinet (POL-EKO Aparatura, Wodzislaw Slaski, Poland) with a 12 h:12 h L:D photoperiod, in which the temperature was set to either 25 °C during isoline production or 20.5 °C to subsequently maintain established isolines for the experiment. A large open container with water placed in the thermal cabinet ensured that the humidity inside the cabinet remained stable at approximately 70%. The flies were kept in fly vials (40 mL; 2.5 cm diameter, 9.5 cm height; polyurethane foam plugs) with 10 mL of cornmeal yeast medium (Bloomington Drosophila Stock Center, Bloomington, USA). We performed regular transfers of flies to new vials with fresh food (every 2 or 3 weeks at 25 °C vs. 20.5 °C), which prevented generational overlap.

### 2.2. Isolines

To establish isolines, each field-collected female was individually placed in a 40 mL vial with food for egg laying. Then, we performed regular transfers of sib-mated females to new vials to obtain new generations, until reaching the 29th generation of highly inbred flies. Upon each transfer, we collected 3 females per line, placing them individually in fresh vials for egg laying for 5 days. Upon the emergence of new flies, we visually checked the abundance and morphology of the new-generation flies, deciding which vial with newly emerged flies (one out of three vials per line) would found the next generation. Thus, the isoline production procedure immediately excluded genotypes that were likely to show negative effects of inbreeding. To ultimately reduce the genetic variance within each line, and thus fix the representative genotypes of each line, the establishment of isolines ended with the controlled mating of virgin females with single brothers. The mates originated from the 29th generation of our highly inbred flies. Multiple mating pairs per line were formed, and each pair was placed in an individual vial with food for mating and egg laying for 48 h. After the emergence of the new generation (30th generation), we chose one vial per line with the most abundant and vigorous flies as the source of parents for the next generation. To generate the subsequent generations (from the 31st onward), upon each transfer, we placed 20 flies from the parental generation in a new vial for mating and egg laying for 5 days, always maintaining 2 backup vials per isoline. Starting from the 32nd generation, the isolines were moved to 20.5 °C (our standard stock environmental temperature), which slowed the transfer intensity. Ultimately, our inbreeding procedure resulted in 15 viable isolines, which were used in this study for the induction of phenotypic changes by rapamycin.

### 2.3. Induction of Two Phenotypes

Each isoline of flies was used to produce two phenotypes by rearing larvae on food with or without rapamycin. Before phenotypic induction, we performed two fly transfers with controlled mating and egg laying with the aim of boosting the number of flies available for the experiment and minimizing the variance in crowding effects. Both transfers were performed under stock conditions, and they involved multiple vials per isoline, with ten females and five males placed in each new vial with fresh food for mating and egg laying for 48 h. The first transfer resulted in 4 vials per isoline, and this generation of flies was reared on standard food. These flies were then allowed to lay eggs under our two experimental conditions (two types of food), such that the second-generation flies from each isoline underwent development on either standard food (control flies) or rapamycin-supplemented food (rapamycin flies). Following Wang et al. [68], the food used for rapamycin treatment was prepared by dissolving rapamycin (Alfa Aesar by Thermo Fisher Scientific, Kandel, Germany) in ethanol (Linegal Chemicals, Warszawa, Poland) and mixing the solution with freshly cooked standard fly food at a 1 μM concentration. The food for the control flies was prepared in exactly the same manner as the food for the rapamycin flies, but was mixed with ethanol alone. Upon the second transfer, five vials with flies per isoline were set up for each developmental treatment. The induction of phenotypes (expecting large cells in the control and small cells in rapamycin treatment) was performed in 40 mL vials with 10 mL of food. Adult males emerging from our developmental treatments were used to study flight performance and morphology. Given the time requirements of flight performance measurements, the induction of phenotypes was distributed over time, performing 4 runs (3 weeks between two consecutive runs) with 3–5 isolines used per run.

### 2.4. Flight Performance

To assess flight performance, we measured the wing-beat frequency in 10- and 25-day-old males originating from the control and rapamycin flies. Our purpose was to expose flies that developed under 20.5 °C normoxic conditions to either mildly or heavily increased metabolic demands during flight (temperature effect) under two different oxygen conditions (oxygen supplies). Our warm and hot environments were represented by two temperatures, 24 °C and 29 °C, which were combined with either normoxia (21% O_2_) or hypoxia (10% O_2_). Two males per isoline in each age group (from either control or rapamycin flies) were measured in each type of environment (32 flies per isoline in total; 480 flies for all groups). To obtain same-age groups, vials with developing control and rapamycin flies were checked daily, and emerging adults were either discarded (if few) or collected (if many) using brief cold anesthesia to obtain males for the measurements. This collection procedure also allowed us to estimate the duration of development, which was defined as the number of days from egg laying (the day of parental flies transfer to vials was counted as day 0) until the emergence of the first adults in a vial. Note that this measure only approximates the developmental duration, as it does not provide detailed information about the emergence dynamics of all flies in a vial. The males collected for the flight performance measurements were placed in new vials with standard food (no rapamycin supplementation of adults). They were kept in these vials until measurements were performed when they reached the ages of 10 and 25 days from emergence. To avoid overcrowding, a maximum of 30 males were placed in each 68 mL vial. To maintain high-quality living conditions, all males awaiting measurements were transferred to new vials with food every 7 days.

Measurements of the wing-beat frequency (Hz) were carried out on tethered flies with the help of an optical frequency counter (OFC) designed by Prodromus (Krakow, Poland) (see the scheme in Figure 1 in [22]). In principle, the OFC collects the same type of data as tachometers used by earlier studies [69], but it uses a different technique. The OFC consisted of a small chamber with thick aluminum walls covered (chamber interior) with insulating foam, allowing the isolation of a fly from ambient conditions (light, temperature, air oxygen concentration) during measurement. For this measurement, a tethered fly was placed in the center of the chamber in the light beam produced by a light-emitting diode mounted at one end of the chamber and collected by the optical collimator at the other end of the chamber. The collimator focused the light beam on a sensor detecting high-frequency light flux disturbances. If necessary, the position of the collimator was adjusted in relation to the fly and the sensor to obtain a sharp image of the fly on the sensor. The wing-beat frequency was recorded 20 times per second, and data were automatically saved. The oxygen and thermal conditions in the measuring chamber were maintained by the constant inflow (5000 mL per minute) of a predefined mixture of oxygen and nitrogen gases by tubing connected to cylinders with either normoxic (21% O_2_) or hypoxic (10% O_2_) gas mixtures (Gaz Centrum, Krakow, Poland). The tubing was connected to a flowmeter that was used to set and monitor the rate of gas flow. Before entering the measuring chamber, the gas mixture travelled through a humidifier and then through a heating/cooling unit, which regulated the temperature of the flow-through gases according to temperature readings received from sensors that constantly monitored the temperature inside the measuring chamber.

The temperature of the inflowing gas mixture in the close vicinity of the tethered fly was independently recorded (°C with ±0.05 precision) with the help of a fast-response thermocouple (0.5 mm diameter) (ACSE, Krakow, Poland) connected to a temperature recorder (Delta OHM, Padova, Italy). Thus, we were able to evaluate that flies were effectively exposed to mean temperatures equal to either 23.8 °C (warm) or 29.1 °C (hot), which were very close to the expected temperature values (24 °C and 29 °C). Immediately before each measurement, we cold-anaesthetized each fly very briefly in preparation for tethering. With the help of UV glue, we attached a thin entomological pin to the upper part of the thorax, and the thus-tethered fly was mounted in the center of an aluminum ring. The ring with the fly was then mounted between the walls of the measuring chamber. Before placing the ring in the measuring chamber, the light beam generated by the OFC was dimmed, and it was brightened to the maximum just before starting the recording. After the measurement, the fly was released from the pin, frozen and then stored for morphological measurements. The flight performance recording lasted 3 min. The first recorded minute was considered to involve habituation to the measurement conditions, so it was discarded from the final analysis. We used a macro designed in the Visual Basic programming language embedded in the Excel program (Microsoft, Redmond, WA, USA) to find the ten-second-long interval in each recording characterized by the highest value of the mean wing-beat frequency (Hz). We considered this value to characterize the maximum flight performance of a fly and used it in our hypothesis testing.

### 2.5. Morphological Measurements

**Figure 1:**
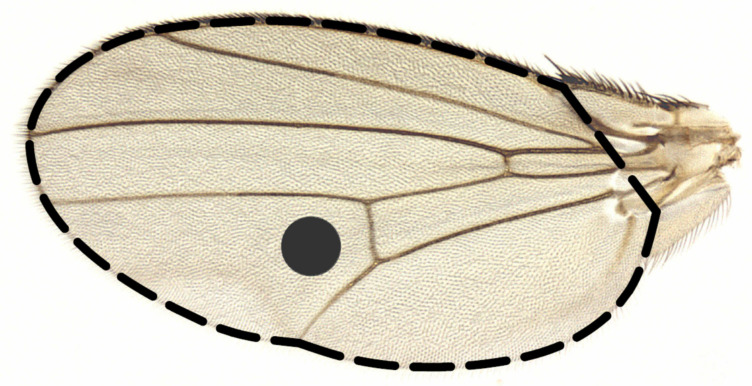
The wing of *D. melanogaster*, showing the region (grey circle) used for counting the trichomes and estimating the size of epidermal cells and the limits (dashed line) of the measurement of wing area.

To assess the effects of rapamycin on fly morphological characteristics, we used the freeze-preserved bodies of males previously subjected to wing-beat measurements. We measured thorax length, wing size, wing load and wing epidermal cell size. Thorax length was measured in all preserved males. After the removal of the left wing, each male was placed on its right side. We measured thorax length to the nearest 0.02 mm as the distance on the thorax from the neck edge to the tip of the scutellum in a left-side view under a stereomicroscope (Olympus Corporation, Tokyo, Japan). Other measurements (wing size, wing load and cell size) were taken from a representative sample of the preserved males (60 males in total), including two males per isoline with each phenotype (control and rapamycin flies). All of these measurements were based on the left wings, which were mounted on microscopy slides using ST Ultra and CV Ultra (Leica Biosystems, Nussloch, Germany). The wings were detached from the body under a stereoscopic microscope using microsurgery forceps to ensure that the cuts were made as close to the thorax as possible. The wing area and cells were measured from digital images of the wings. Images for wing area measurements were taken with OPTA View software (OPTA-TECH, Warsaw, Poland) and a stereomicroscope (Nicon Corporation, Tokyo, Japan) equipped with a camera (OPTA-TECH, Warsaw, Poland). To estimate wing area (mm^2^), we outlined the entire wing (Figure 1) from the costal cell to the alula using ImageJ software with a LiveWire Plugin (National Institution of Health, Bethesda, USA). Images for cell size measurements were taken under higher magnification with ZEN 2011 software (ZEISS, Oberkochen, Germany) with the help of a stereomicroscope (Nicon Corporation, Tokyo, Japan) equipped with a camera (Nicon Corporation, Tokyo, Japan). According to Dobzhansky [70], one *Drosophila* wing epidermal cell produces one trichome. This allowed us to estimate the mean size of epidermal cells from the density of trichomes in a fixed area of a wing. Trichomes were counted on the dorsal wing blade in a 0.03 mm^2^ circle placed on the wing between the cubital and distal veins (Figure 1) [47]. Counting was carried out automatically by two macros embedded in ImageJ software. The first macro converted the photo into a binary image by extracting a green RGB channel from it. After this step, we checked the photos, manually wiped out trichomes that were rooted outside the circle and separated the interconnecting trichomes. Then, using the second macro, we obtained the number of trichomes within the circle. To calculate the mean cell size (µm^2^), the area of the circle was divided by the number of corresponding trichomes. The wing load was defined as follows: thorax length^3^·wing area^−1^ (mm^3^·mm^−2^) [71,72].

### 2.6. Data Analysis

We analyzed the data with general linear mixed modelling (GLMM), which was performed in R 4.0.3 software [73] with the help of lme4 [74], lmerTest [75] and car [76]. Graphics were prepared with the ggplot2 [77] and emmeans packages [78]. Prior to the analysis, data on wing-beat frequency and thorax length were cube transformed. In the first step, we examined the effects of rapamycin supplementation on larval development and adult morphological traits. For this purpose, we used a set of GLMMs to analyze data on the duration of development, thorax length, wing load and cell size. Each model considered the phenotype (rapamycin vs. control flies) as a fixed predictor and the time block (a run of phenotypic induction) and the isoline (15 isolines) nested in the time block as two random effects. Note that the data on thorax length came from all individuals subjected to wing-beat measurements (N = 480), while the data on wing load and cell size came from only a subset of the flies subjected to wing measurements (N = 60). Additionally, the data on developmental durations were represented by a single value characterizing either the control or rapamycin flies of each isoline (N = 30). In the second step, we examined whether flight performance differed between the two phenotypes (control vs. rapamycin flies), flight conditions (two thermal environments combined with two oxygen conditions) and different fly ages. For this purpose, we used a GLMM to analyze our measure of the greatest flight performance. The model considered the phenotype (control vs. rapamycin flies), thermal conditions during measurements (warm vs. hot), oxygen conditions during measurements (normoxia vs. hypoxia) and age (10 vs. 25 days) as fixed grouping predictors. The time block and id of the isolines (nested in the block) were considered random effects. The model also included a 3-way interaction between thermal conditions (warm vs. hot), oxygen conditions (normoxia vs. hypoxia) and the cell-size phenotype (control vs. rapamycin flies). This interaction helped us to test whether the flight performance of the small-cell vs. large-cell-phenotype flies was similarly limited by hypoxia and whether this limitation was similarly pronounced under each thermal condition. See Supplementary Materials.

## 3. Results

**Figure 2:**
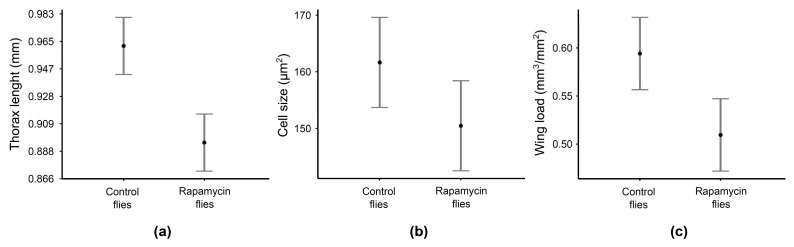
Adult males of *D. melanogaster* that developed on food supplemented with rapamycin (rapamycin flies) had smaller thoraxes (**a**), smaller wing epidermal cells (**b**) and a lower wing load (**c**) than flies that developed on standard food (control flies). The graphs show means with 95% confidence intervals estimated from a statistical model. Data on thorax length were transformed back to the original values for the purpose of generating this graph to make it easier to read the actual values.

GLMM analysis showed that rapamycin supplementation of *D. melanogaster* larvae prolonged their development (by 13.8%; F = 42.25, *P* < 0.0001) and produced a distinct fly phenotype. Adult males treated with rapamycin were characterized by smaller thoraxes (by 7.2%; F = 584.42, *P* < 0.0001; Figure 2a), smaller wing epidermal cells (by 6.9%; F = 33.08, *P* < 0.0001; Figure 2b) and a lower wing load (by 14.2%; F = 68.25, *P* < 0.0001; Figure 2c) than the control males.

**Figure 3:**
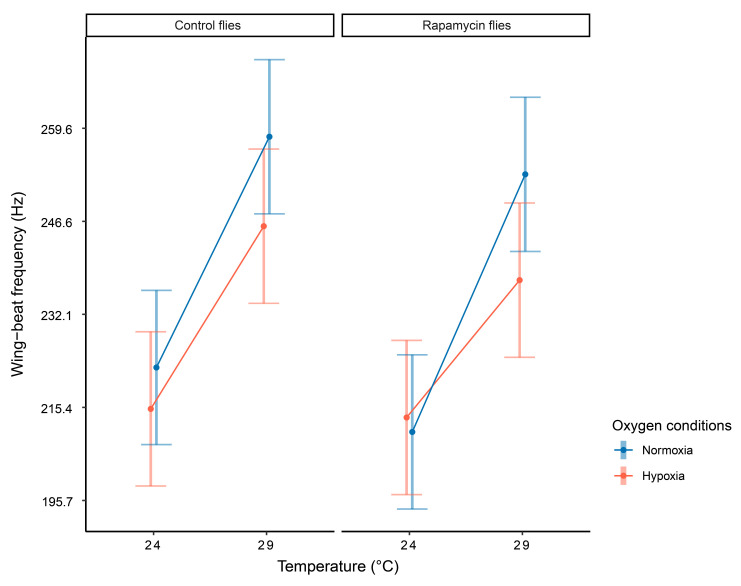
The effects of temperature and oxygen conditions on flight performance differed between adult males of *D. melanogaster* originating from two different developmental conditions (standard food (control flies) vs. standard food supplemented with rapamycin (rapamycin flies)). All tested flies underwent development at 20.5 °C. When the flies were exposed to more metabolically demanding thermal conditions (hot, 29 °C) and their wing-beat frequency increased, hypoxia slowed flight performance equally in the control (large cells) and rapamycin (small cells) flies. However, when flies were exposed to less demanding thermal conditions (warm, 24 °C) and their wing-beat frequency was lower, oxygen retardation was only observed in the control flies, whereas the rapamycin flies did not respond to the oxygen level. The graph shows the 3-way interaction with means and 95% confidence intervals estimated from a statistical model (see Table 1 for model details). Data on wing-beat frequency were back transformed for the purpose of generating this graph to make it easier to read the actual values.

The analysis of the wing-beat frequencies of the flies with the two different phenotypes (Table 1) generally showed significant effects of the conditions during the measurements (temperature and oxygen) and dependence of these effects on the fly phenotype but no effect of fly age. The flies flapped their wings at higher speeds in conditions with a higher temperature (F = 77.63, *P* < 0.0001) or a higher oxygen concentration (F = 13.10, *P* < 0.028), although the effect size of temperature was much greater (Figure 3). Our GLMM showed a significant interaction between the thermal and oxygen conditions during the measurements and the cell-size phenotype (F = 2.59, *P* < 0.035). This indicated that the effects of temperature and oxygen on flying *Drosophila* could not be fully interpreted without the simultaneous consideration of the phenotypic effects of our developmental treatments (control vs. rapamycin). As shown in Figure 3, when flies were exposed to less severe heat (our warm condition), hypoxia retarded flight performance only in the control flies (large cells), whereas the rapamycin flies (small cells) flapped their wings at comparable frequencies irrespective of the oxygen conditions. However, when the flies were exposed to more severe heat (our hot conditions), under which the demand for oxygen was very high and the flies were far beyond their acclimation temperatures, the retardation of flight performance associated with the oxygen supply was observed in the control and rapamycin flies. Moreover, Figure 3 indicates that while wing beat frequency consistently increased with temperature in all flies, the hypoxic rapamycin flies were characterized by the weakest thermal response among all groups of flies.

## 4. Discussion

The results of our study demonstrated that rapamycin supplementation of developing *D. melanogaster* larvae resulted in the emergence of adult flies with smaller wing epidermal cells. Our experimental conditions clearly produced the cell-size phenotype that was needed for our further exploration of the links between cell size and flight performance (see next part of Discussion). These results agree with the current understanding of how TOR activity during development affects the growth and proliferation of cells (e.g., [43,54]). It is worth noting that this knowledge largely comes from research on organisms with genetically engineered changes in different TOR elements, with a considerable contribution from studies of *D. melanogaster* TOR mutants [58,59]. In accordance with the conclusions of genetically based research, studies of environmentally induced changes in TOR have shown that rapamycin-induced downregulation of TOR activity results in smaller cells [79,80,81,82]. However, this evidence has originated almost exclusively from studies of cell cultures of many different organisms (e.g., from fruit flies, mice, rats and humans). Our study apparently fills an important gap in the research, providing evidence that the supplementation of developing organisms with rapamycin and, thus, the downregulation of TOR activity during development at an organism-wide level leads to changes in the cell size cycle and produces adults with smaller cell sizes. Previously, Wu et al. [83] demonstrated that provisioning *Drosophila* larvae with 5 and 10 µM rapamycin reduced cell size in the wings of adult males by 8% and 10%, respectively, but this supplementation was carried out only during the last 24 h of larval development (at 25 °C). We applied smaller amounts of rapamycin (1 µM) during the entire larval development process (at 20.5 °C), which resulted in a ca. 7% reduction of cell size. Studies of temperature-induced changes in the cell size of *D. melanogaster* suggest that the sooner developing larvae experience thermal conditions that can change cell size, the more pronounced the cell size changes will become in adults [84], suggesting that cell-size control mechanisms are more sensitive to environmental triggers early in development. Nevertheless, the developmental stage at which the rapamycin-induced alteration of TOR activity results in the most pronounced cell-size changes in adults should be further explored. This information will provide hints about the time windows during development in which TOR activity determines the cellular architecture of adult stages.

Our results offer an opportunity to further explore the effects of TOR activity on some fitness-related morphological and life history parameters. In addition to the effects on cell size, the inhibition of TOR by rapamycin prolonged larval development and resulted in smaller bodies of adult flies, which were also characterized by relatively larger wings and, thus, a lower wing load during flight. Longer development and late eclosion of flies growing on media containing rapamycin was previously reported by Oldham et al. [58], Zhang et al. [59], Potter et al. [85] and Scott et al. [86], but to our knowledge, the effects of rapamycin supplementation on adult body size with reference to wing load have not previously been studied, especially in light of the cellular mechanisms of organ size changes. In ectotherms, including insects, the adaptive value of life history responses to environmental parameters, such as thermal conditions, is a subject of ongoing research and scientific debate [87]. Adult body size significantly affects Darwinian fitness and shows dramatic changes on different biological scales, either evolving among populations and species or changing plastically when genotypes respond to gradients of developmental conditions [6,43,88]. For example, ectotherms reared in warmer conditions tend to grow faster and mature earlier and at smaller body sizes, leading to an inverse correlation between environmental temperatures and body size; this phenotypically plastic response is referred to as the temperature–size rule (TSR) [89]. On a large geographic scale, studies comparing different populations have reported that small-body ectotherms often occur in warmer habitats (e.g., in low-latitude locations) [36,90,91,92], a clinal pattern similar to Bergmann’s rule originally described for endotherms [93]. At a cellular level, differences in body size among organisms, such as those addressed by TSR and Bergmann’s rule, can arise via alterations in cell size or cell number, or, more likely, involving both mechanisms simultaneously. Our results clearly demonstrate that the inhibition of TOR activity resulted in combined changes in body size and cell size, such that smaller adult flies were characterized by smaller cells in their body. This correlated plastic response of body size and cell size resembles developmental responses of ectotherms to oxygen [94,95], food [96] and thermal conditions ([36,37,41,92,97,98], but see [30,35,99] for more complex patterns of the thermal dependence of cell size and body size in ectotherms). Addressing the concerted changes in cell size and body size is also relevant for studies aimed at body size differences among taxa [43]. For example, an interspecific comparison of Hawaiian *Drosophila* demonstrated that the origin of larger species involved an increase in cell size in different organs [100]. Similarly, Schramm et al. [56] showed that the origin of larger species or a larger sex in carabid beetles involved cell size increases in different cell types. The contribution of cell size changes to interspecific differences in body mass was also shown by phylogenetically informed comparisons of amphibians, birds and mammals [101] and rodents and galliform birds [31].

Our results showed that rapamycin-induced reductions in cell size and body size were accompanied by changes in wing size, which resulted in a lower wing load. Wing load is another trait of insects that varies not only within populations [102] but also among populations distributed along environmental gradients [12] and influences insect flight [71]. A lower wing load has been proposed to confer an adaptive advantage in cold environments [72,103] as it lowers power requirements and can aid in lift production [104]. Thus, a lower wing load is often viewed as beneficial in challenging flight conditions, especially at high elevations [12]. Indeed, Frazier et al. [102] demonstrated that flies reared at 15 °C showed increased wing dimensions relative to their body size and, thus, a decreased wing load, which improved flight performance at lower temperatures. However, Dillon and Frazier [12] did not show effects of wing load on the flight performance of flies. Interestingly, the relationship between the body size of flies and wing load looks different if we focus on the within-population differences in body size (flies that develop under similar conditions) or on across-population differences (e.g., manifest effects of different thermal environments). At the within-population level, small individual flies are characterized by a decreased wing load [12,102], but small flies that result from warmer developmental conditions (TSR) are characterized by an increased wing load [12,102,105]. From this perspective, the combined effects of rapamycin supplementation (decreased body size and wing load) seem to resemble the within-population variance of flies rather than the thermally driven responses.

There is some evidence suggesting that it can be more challenging for insects to meet their oxygen demands in warmer environments [20,106], but this effect is unlikely to occur in terrestrial insects at rest. Our results showed that an acute change in air temperature or air oxygen content affected the flight performance of flies, but these patterns further depended on the cell-size phenotype of the flies. Generally, the flies showed a higher maximal wing-beat frequency at higher air temperatures (hot vs. warm conditions), which is in accord with textbook expectations for the thermal dependence of an ectotherm [87] and agrees with previous studies of flight performance in *D. melanogaster* (e.g., [102,107]). We note that the thermal dependence of insect flight has been much less frequently studied than the thermal dependence of other insect traits (see, e.g., [87]). The body temperature of an insect strongly affects the dynamics of its muscle contractions and physiology and its metabolic rate in general; hence, it is also considered the major determinant of flight performance [12,26]. It is well established that large insects, such as some moths [108], bees [109] and syrphid flies [110], often show preflight warm-up activities aimed at activating ventilatory mechanisms and increasing thoracic temperatures and metabolic rates, thus helping to initiate flight [111]. Importantly, we found evidence that a decreased oxygen content of the air can impose a significant challenge to the tracheal system of insects in delivering oxygen to the mitochondria in flight muscles. The flies exposed to our hypoxic condition (10% O_2_) generally showed a lower maximal wing-beat frequency than the flies exposed to normoxia (21% O_2_). Similarly, earlier studies have reported that fruit flies [22,112], honeybees [113], dragonflies [114,115] and locusts [116] exhibited decreased flight performance during acute exposures to conditions with lower oxygen availability.

Our hypoxic conditions limited the flight performance of the control flies (large-cell phenotype) under both warm (~24 °C) and hot (~29 °C) thermal conditions. In contrast, the rapamycin flies (small-cell phenotype) showed flight limitation by hypoxia only in our hot environment, whereas they flapped their wings at equal frequencies irrespective of oxygen conditions in the cooler (warm) environment. Moreover, our results suggest that while wing-beat frequency consistently increased with temperature in all flies, the rapamycin flies exposed to hypoxia showed the weakest thermal response. To fully understand this pattern, let us first note again that prior to the flight measurements, all flies were developmentally acclimated to a 20.5 °C temperature. Therefore, both thermal treatments applied during the flight measurements exposed flies at flight to warmer conditions, creating an acclimation mismatch during highly metabolically demanding activity. However, our warm treatment (24 °C) was much closer to the acclimation temperature (20.5 °C) than the hot treatment (29 °C). Moreover, given the evidence of the thermal sensitivity of fitness-related activities of *D. melanogaster* [117,118,119,120,121], the temperature close to 24 °C in our warm treatment was likely to be close to the thermal performance optimum of this species. In fact, temperatures of 25 °C are routinely used to maintain fly stocks. In contrast, the temperature of 29 °C in our hot treatment was much closer to the upper thermal limits reported for *D. melanogaster* and for many other insects (depending on which type of performance is measured). For example, evidence from different *Drosophila* species, including *D. melanogaster*, showed that the highest flight performance measured as the proportion of successful flights peaked at 24 °C and decreased at 28 °C [122]. Similarly, in the moth *Mamestra brassicae*, flight performance (including the wing-beat frequency) increased from 12 °C to 24 °C, which was followed by a sharp decrease in the range of 24 °C to 32 °C [63]. According to Makumbe et al. [123], the oriental fruit flies *Bactrocera dorsalis* fly the longest distances at temperatures ranging from 20 °C to 24 °C. Notably, our source population originated in a temperate and relatively cool climate (Poland), so it is likely that the flies studied here were not well adapted to perform under more severe heat, at least compared to conspecifics from tropical regions. All things considered, our thermal conditions during testing (warm vs. hot) imposed two types of physiological challenges on flying *Drosophila*, one that was caused by a deviation from the acclimation temperature and another that was caused by a match between each of the two testing conditions and the evolved physiological thermal optimum and thermal limits. Given these two thermal challenges, the results of our experiment suggest that flight performance was consistently limited by hypoxia, irrespective of cell size in the body, when flies were poorly thermally acclimated to their new body temperatures and these temperatures were closer to the physiological thermal limits (our hot condition). In contrast, when flies were more thermally acclimated to their new body temperatures and these temperatures were far from the physiological thermal limits (our warm condition), flight performance was limited by hypoxia only in the large-cell-phenotype flies, whereas the flight of the small-cell-phenotype flies remained insensitive to air oxygen levels. According to TOCS, the relatively large cell membrane surface area of small cells should increase the rate of oxygen fluxes in tissue (as the diffusion of oxygen is faster in lipids than in water), which should result in a better thermal performance of small-cell organisms, especially when oxygen delivery to mitochondria is at risk of limitation (decreased air oxygen availability, metabolically demanding activities). This prediction suggests that increased tolerance of thermal extremes occurs in small-cell organisms. For example, Verspagen et al. [124] showed that *D. melanogaster* flies reared in warmer conditions were characterized by smaller cells and survived longer under acute, intense heat stress (39 °C) than cold-reared flies (with larger cells), which is consistent with the effects of cell size on oxygen delivery to mitochondria predicted by TOCS. Walczyńska et al. [41] demonstrated that small-cell freshwater rotifers presented superior reproduction rates to large-cell rotifers in warm waters under hypoxic conditions, but they were outcompeted by large-cell rotifers, both in cool waters and under normoxic conditions. In contrast, we did not find evidence supporting the connection between *D. melanogaster* cell size and oxygen delivery under thermal conditions approaching physiological thermal limits. Instead, in accord with TOCS, our results suggest that small cells can increase the rate of oxygen delivery to mitochondria under more thermally benign conditions that are closer to physiological optima than to thermal limits. In fact, insects are likely to behaviorally regulate their body temperatures in nature, e.g., by adjusting their daily activity to thermal conditions [125] or choosing thermally optimal microhabitats [126], thus avoiding direct exposure to extreme heats. This perspective increases the ecological relevance of our results, which demonstrated links between cell size and flight sensitivity to the oxygen supply, but only under benign thermal conditions. TOCS also suggests another phenomenon that helps to understand our results—organs that consist of small cells can confer benefits due to their internal high transport capacity created by the large total cell surface area and short distances within cells. Nevertheless, the relatively large amount of cell membrane imposes costs associated with maintaining ion gradients at the cell surface [40,42,47]. For ectotherms exposed to thermal fluctuations, another type of metabolic cost results from maintaining the physical integrity of cells, which involves constant rebuilding of membranes to restore the optimal physical state following a thermal change [37,39,47], which is referred to as the homeoviscous adaptation of membranes [127]. If so, small-cell phenotypes characterized by relatively large amounts of cell membranes might be more challenged by acclimation needs than large-cell phenotypes, especially during highly metabolically demanding activities, such as flight. This would account for the superior capacity of the small-cell flies to cope with hypoxic conditions under the warm treatment applied herein and the loss of this capacity under hot conditions, which would require intense homeoviscous adaptation, especially in the small-cell-phenotype flies. Certainly, this hypothesis requires rigorous testing, especially because some previous studies showed that thermal fluctuations during larval development resulted in adult *Drosophila* flies with reduced cell size [37,47].

In addition to examining the thermal dependence and oxygen limitation of insect performance, many earlier studies have used insects, particularly *Drosophila* flies, as model organisms to address age-dependent changes in organismal performance [128,129,130,131,132]. Ageing-oriented studies that have focused on different measurements of insect flight performance, such as flight duration, flight speed, flown distance, flight initiation or wing-beat frequency, have reported inconsistent age-related patterns [61,62,63,64,65,123,133]. Moreover, the origin of such patterns seems to also depend on past flight experience, as found at least in *Drosophila* flies [134]. This complex picture of age-related changes in the flight performance of insects indicates that we are far from a full understanding of ageing processes, especially with respect to flight capacity. Our comparison of younger and older flies (10 vs. 25 days after eclosion) adds to this discussion, as we found no measurable differences in the maximal wing-beat frequency between our two age groups, indicating that this aspect of flight performance does not deteriorate drastically with insect age. In support of this hypothesis, Privalova et al. [22] studied ageing patterns in more detail in the locomotion of *D. melanogaster*, reporting no systematic deterioration in the maximal wing-beat frequency with ageing, although the flies were monitored much longer than in our study, until the age of 50 days post eclosion. At the same time, Privalova et al. [22] reported that their studied flies showed a systematic decrease in climbing capacity, another type of locomotory performance, which indicated that physiologically old flies were still able to flap their wings at frequencies comparable to their much younger conspecifics. In accord with these results, no effects of ageing on flight capacity were observed in brown marmorated stink bugs, *Halyomorpha halys* (measurements conducted until 47 days of adult life) [67]. On the other hand, Miller et al. [66] demonstrated the inability of *D. melanogaster* flies to beat their wings on the 56th day of adult life (considered old flies). Overall, the capacity of insects to rapidly deliver oxygen to their flight muscles seems to be little affected by age, which may indicate that the maintenance of flight muscle performance remains under especially strong selective pressure in flying insects [22]. Moreover, the inconsistent age-related patterns of flight performance vs. other types of locomotory performance (e.g., climbing or walking) may be rooted in different physiologies of contractions between the muscles involved in flight and in other types of locomotion [22].

## 5. Conclusions

Here, we demonstrated that rapamycin supplementation of larvae and, thus, the downregulation of TOR signaling pathways during development resulted in smaller adult flies with smaller cells in the body. Importantly, we showed that while flying flies generally slowed their wing strokes in cooler or less oxygenated air, flies with smaller body cells (rapamycin supplementation) were less prone to oxygen limitation, especially when the environmental temperature better matched the thermal optimum of the flies for physiological performance and their acclimation temperatures. Following TOCS, we suggest that ectotherms with small-cell life strategies can maintain superior performance during metabolically demanding activities (e.g., flight) when challenged by oxygen-poor conditions, but this advantage may depend on the correspondence among body temperature, thermal acclimation and physiological thermal limits. Like many other organisms, insects commonly experience daily and seasonal thermal fluctuations [102], but among the different insect responses to these fluctuations [135], changes in flight performance have rarely been studied. Thermal and oxygen conditions also vary on a geological time scale and a geographic scale (e.g., latitudinal and elevational gradients), so our results help to better understand the selective pressures imposed on flying insects by spatiotemporal environmental gradients. Thus, our study enters the discussion of the biological consequences of anthropogenic environmental changes, which involve not only the effects of rising global mean temperatures but also the effects of the increased frequency of locally appearing heat waves or heat islands established by urban activities [136,137,138,139,140]. To date, mainstream research addressing human impacts on ectotherms has focused on connections between environmental changes and species’ geographic distributions, survival and body sizes (e.g., [135,141,142,143,144,145,146]), but our study suggests that this perspective should also include the connections between cell-size life strategies and organismal performance in the changing world.

## Figures and Tables

**Table 1:** Results of a general linear mixed model of wing-beat frequency in two cell-size phenotypes of *Drosophila melanogaster* males (developed at a common temperature (20.5 °C) on either food supplemented with rapamycin (small cells) or food without rapamycin (large cells)). Flight performance was measured in two age classes (males aged 10 and 25 days) in four types of conditions: two elevated temperatures (warm, 24 °C and hot, 29 °C) combined with two oxygen levels (normoxia and hypoxia).

Effect	F	Df	*P*
Temperature (warm vs. hot)	77.63	1	<0.0001
Phenotype (small cells vs. large cells)	1.65	1	0.199
Oxygen (normoxia vs. hypoxia)	13.10	1	0.028
Age (10 vs. 25 days)	0.97	1	0.324
Temperature × oxygen × phenotype	10.37	1	0.035

## Data Availability

The data presented in this study are available in the Supplementary Materials.

## Supplementary Materials

The following Supplementary Dataset is available online at https://www.mdpi.com/article/10.3390/biology10090861/s1.
